# Rural Land Use Change during 1986–2002 in Lijiang, China, Based on Remote Sensing and GIS Data

**DOI:** 10.3390/s8128201

**Published:** 2008-12-11

**Authors:** Jian Peng, Jiansheng Wu, He Yin, Zhengguo Li, Qing Chang, Tianlong Mu

**Affiliations:** 1 Key Laboratory for Environmental and Urban Sciences, Shenzhen Graduate School, Peking University, Shenzhen 518055, P.R. China; 2 College of Urban and Environmental Sciences, Peking University, Beijing 100871, P.R. China; 3 Key Laboratory of Resources Remote Sensing & Digital Agriculture, Ministry of Agriculture, Beijing, 100081, P.R. China; 4 Department of Ornamental Horticulture and Landscape Architecture, China Agricultural University, Beijing 100094, P.R. China

**Keywords:** Rural land use change, Spatial patterns, Change rate, Remote sensing, GIS, Lijiang, China

## Abstract

As a local environmental issue with global importance, land use/land cover change (LUCC) has always been one of the key issues in geography and environmental studies with the expansion of regional case studies. While most of LUCC studies in China have focused on urban land use change, meanwhile, compared with the rapid change of urban land use in the coastal areas of eastern China, slow but distinct rural land use changes have also occurred in the mountainous areas of western China since the late 1980s. In this case through a study in Lijiang County of Yunnan Province, with the application of remote sensing data and geographic information system techniques, the process of rural land use change in mountain areas of western China was monitored through extensive statistical analysis of detailed regional data. The results showed significant increases in construction land, paddy field and dry land, and a decrease in dense forest land and waste grassland between 1986 and 2002. The conversions between dense forest land and sparse forest land, grassland, waste grassland and dry land were the primary processes of rural land use change. Sparse forest land had the highest rate of land use change, with glacier or snow-capped land the lowest; while human settlement and rural economic development were found to be the main driving forces of regional difference in the integrated land use change rate among the 24 towns of Lijiang County. Quantified through landscape metrics, spatial patterns of rural land use change were represented as an increase in landscape diversity and landscape fragmentation, and the regularization of patch shapes, suggesting the intensification of human disturbances and degradation of ecological quality in the rural landscape.

## 1. Introduction

Both remote sensing (RS) and geographic information system (GIS) have been widely used in the fields of global/regional environmental change, especially when the spatial dimension is considered in the studies. Using RS, spatially explicit time series of environmental data can be quickly obtained and updated [1, 2], with GIS techniques providing the software to spatially analyze, model and map environmental change. Therefore, RS and GIS have been recognized as powerful and effective tools in monitoring environmental change at broad scales, especially in detecting the land use/land cover change (LUCC) [3-10].

Since the LUCC plan was jointly presented by International Geosphere and Biosphere Programme (IGBP) and International Human Dimension Programme (IHDP) in 1995, as a local environmental issue with global importance [11], LUCC has always been one of the key issues in geography and environmental studies [12]. Currently, the focus on LUCC includes the monitoring and mapping of land use change, the analyzing of driving forces, the modeling and predicting of land use change with different scenarios, and the assessing of ecological effects associated with land use change. All the fields make up of a full LUCC research, among which the detecting of land use change is of great importance, as it is the first step to provide primary data for the latter studies in a full LUCC research. Based on remote sensing and GIS, numerous case studies at multiple spatio-temporal scales have been conducted to detect different types of land use change in such representative areas as coastal zones [9, 10, 13-18], metropolitan areas [2, 6, 19-23], fast urbanizing areas [4, 5, 24-32], agricultural areas [8, 33-38], arid or semi-arid areas [39-45], hilly areas [46-48], wetlands [49-52], and mining areas [3, 53]. In these studies, various methods were developed for change detection, among which the post-classification comparison approach was widely used to monitor land use change with the application of time series of remote sensing images [10, 22, 54-56].

Generally speaking, land use change means the conversion among land use types in a landscape which not only leads to the change of area ratio of land use types, i.e. land use structure, but also results in the dynamics of the configuration and proximity of landscape patches, i.e. land use patterns. The change of land use structure has been mainly conducted through detecting the change extent and rate, and the conversion loss or gain of each land use type; while to reveal the characteristics and dynamics of landscape spatial patterns, the set of landscape metrics should include at least three aspects: shape characteristics of individual landscape patches, spatial configuration of landscape elements, and diversity of the entire landscape. However, at present, far more attention has been paid to the change of quantitative structure, and only a few are concerned with the change of spatial patterns [7, 10, 23, 39, 57]. Even in the studies of the change of land use structure, few are focused on the rate of land use change [58, 59]. Therefore, it can be concluded that, until now few studies have made an integrated analysis on the change of land use structure and spatial patterns, with a concentration on land use change rate.

In addition, as urbanization has been a growing global trend since the 20^th^ century [60], LUCC in highly urbanized and fast urbanizing areas has attracted much more interest among a wide variety of studies [31], especially in China, a country experiencing unprecedented and accelerated urbanization since the 1980s [61]. In contrast with the flourishing studies on urban sprawl and associated land use change in eastern and coastal China [5, 10, 21-25, 30, 51, 58, 62-65], little research has been concentrated on the processes, the driving forces or the ecological effects of rural land use change in the mountain areas of western China, although there have been distinct quantitative structure and spatial pattern land use changes along with low-speed but efficient rural development and social transformation.

In order to enrich regional cases of global rural land use change in mountain areas of western China, Lijiang County in Northwestern Yunnan Province, a typical rural minority area with fragile environment and low economic development, was chosen as a case study area. More specifically, the aims of this study are to monitor and map rural land use change in Lijiang County during 1986-2002 based on Landsat TM images, to quantify the change extent of land use structure through the calculation of land use change matrix, to compare the change rates among various land use types or townships, and to make a quantitative analysis on the change of land use patterns with the application of landscape metrics.

## 2. Study area

**Figure 1.**
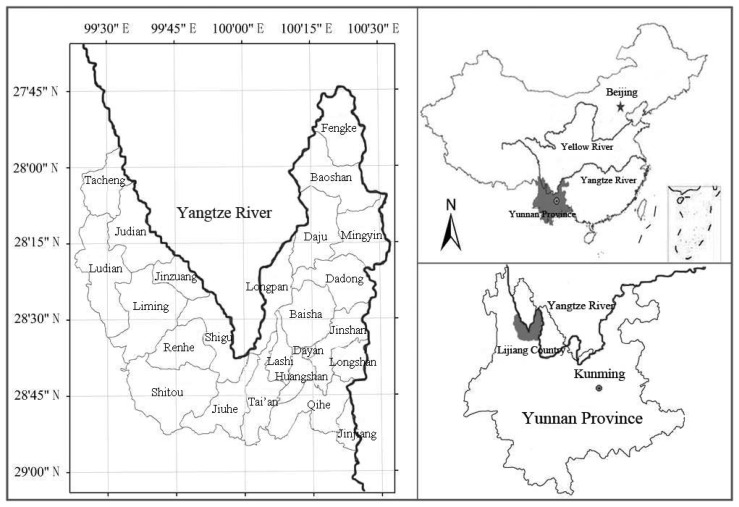
Location of the study area Lijiang County of Yunnan Province, P.R. China.

Lijiang County, one of the most ecologically fragile mountainous areas in China [66], is situated in northwestern Yunnan Province, China. It ranges from 99°23′E to 100°32′E, and from 26°34′N to 27°46′N. The county covers a land area of 7,648 km^2^, with the distances from east to west and from south to north 112 km and 150.7 km, respectively. Lijiang County is located on the upper reaches of the Yangtze River, with about 447 km of the Yangtze River flowing through the county (Figure 1).

Lying in the transition zone extending from the low altitudes of the Yunnan Plateau to the high altitude of the Qinghai-Tibet Plateau, Lijiang County is crisscrossed by rivers and mountains. The whole county is characterized by high mountains and deep gorges, resulting in the rugged topography. The altitude in the study area varies greatly from 1,219 m at Jinsha River to 5,596 m at Yulong Mountain with an average altitude of 2,400 m, with altitude decreasing from northwest to southeast. There are 42 mountains with an altitude above 3,500 m, belonging to the northwest-to-southeast mountain range of Yunling, or the northeast-to-southwest mountain range of Yulong. The two mountain ranges intersect with each other at Shigu Town, resulting in the V shape of the county. The principal landform types are mountain, basin and valley, which cover about 85%, 4.7%, and 10.3% of the county respectively. The mountain monsoon climate influences the study area, with distinct dry and rainy seasons. The mean daily temperature is 12.6°C, with mean annual solar radiation intensity of 146.5 kcal/cm^2^, and mean annual non-frost period of 294 days. The mean annual rainfall is 953.9 mm, with more than 80% concentrating during June to September.

Lijiang County is the exclusive minority autonomous county of the Naxi nation. Until the end of 2002, there were 24 towns in Lijiang County, and the population of the whole county was 350,826, with about 79.78% agricultural population, 83.07% minority population, and 58.54% Naxi ethnic population. Due to its remote location in China and Yunnan Province, mountainous terrain, and scant transportation to the outside, there was negligible economic development in Lijiang County before 1990s. However, because of the beautiful natural landscapes and unique Naxi ethnic culture, Lijiang County has been on the top list of tourism destinations for travelers in China or abroad since the 1990s. In 1997, the Old Town of Lijiang was added to the World Heritage List of Cultural Properties. In 2003, as an important part of Three Parallel Rivers of Yunnan Protected Areas, Laojun Mountain was inscribed on the World Heritage List of Natural Properties, and the Dongba classical documents of the Naxi ethnic group were included in the World Memory Heritage List. Correspondingly, the number of tourist arrivals rose steadily from 0.7 million in 1995 to over 2.9 million in 2001, with tourism income increasing from 160 million RMB to 1,800 million RMB. Associated with rapid development of tourism since 1985, especially after tourism was confirmed as the primary industry of Lijiang County in 1994, there has been accelerated economic growth. In 2002, the Gross Domestic Product (GDP) of Lijiang County amounted to 1.7 billion RMB. However, the economy of Lijiang County is still in the first phase of industrialization, with the economic output of the county far smaller than counties in eastern China.

## 3. Methodology

### 3.1 Data

The analysis of land use change in Lijiang County was based on 16-year time series remote sensing data, which were predominantly cloud-free Landsat Thematic Mapper (TM) images (WRS path 131, row 42; path 132, row 41; path 131, row 41) acquired on November 2, 1986 and February 7, 2002, respectively. The resolutions of the images are 30 meters. In each satellite image, 6 bands, i.e. band1-band5 and band 7, were used as the primary data to monitor land use change of the study area. To unify the geo-reference of subjects, TM images were coordinated in ERDAS 8.4 with the system of Albers Equal Area system with original longitude 105°E and original latitude 0°N, double standard parallel of 25°N and 47°N, Beijing 1954 geodetic datum and Krassovsky ellipsoid [48].

**Figure 2.**
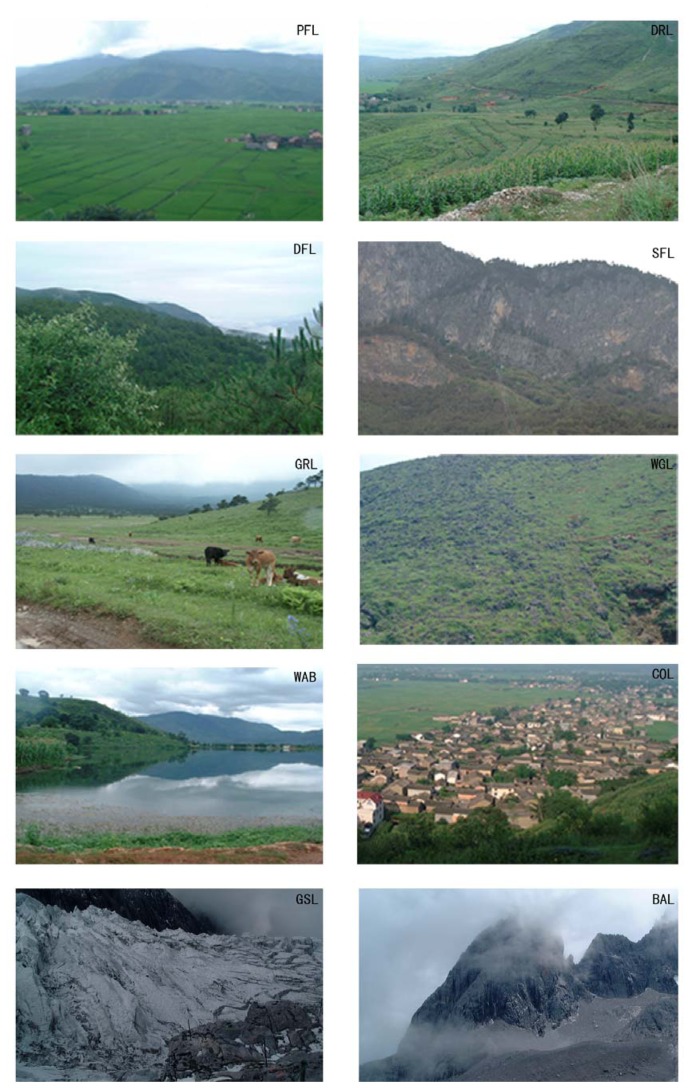
Photos showing the landscapes of different land use types in Lijiang County.

Based on a 1993 land use map at a 1:100,000 scale, two digital elevation maps in 1992 at a 1:250,000 scale and in 2002 at a 1:50,000 scale respectively, and a reconnaissance field surveys in July of 2001, TM imagines were interpreted with the application of supervised classification and visual interpretation, so as to guarantee classification consistency and accuracy. In order to detect the associated ecological effects of rural land use change in detail, land use classification was conducted based on current status of human land use and natural ecosystem types in the study area. In summary, ten land use types were classified (Figure 2): paddy fields (PAF), dry lands (DRL), dense forest lands (DFL), sparse forest lands (SFL), grasslands (GRL), water bodies (WAB), glacier or snow-capped lands (GSL), construction lands (COL), waste grasslands (WGL), and bare lands (BAL).

Among these land use types,

- PAF means irrigated land situated on flat areas, while DRL lacks irrigation water or facilities, relying on natural precipitation;
- DFL refers to the forest land with a coverage density above 30%, while the other forest land was classified as SFL, which includes shrub lands, open woodlands, garden, planted areas with young forest and nurseries;
- GRL means the areas with herbaceous vegetation cover used for grazing, including natural or artificial grassland;
- WGL refers to unused grassland, which usually has a herbage coverage density below 10%;
- WAB included rivers, lakes, reservoirs and ponds, and GSL was covered by permanent glacier or snow in the mountains with an elevation above 4,250 m;
- COL included urban settlements, rural settlements, individual industry and mining land, and transportation and associated green land;
- BAL included bare rock or sand with little vegetation coverage.

**Figure 3.**
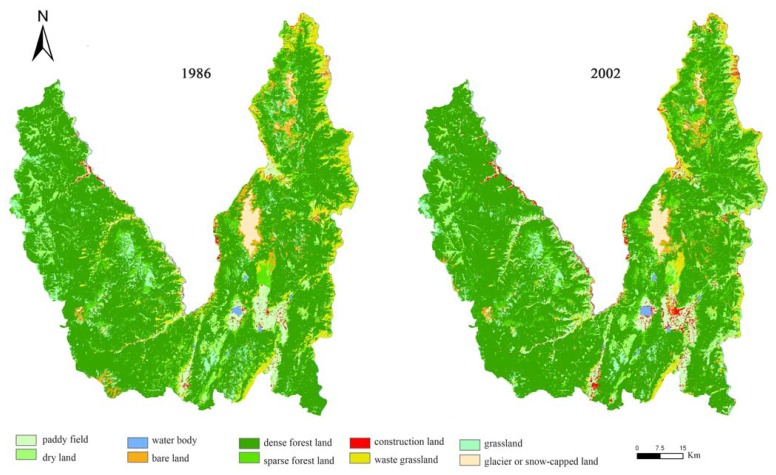
The classification maps of land use in Lijiang County in 1986 and 2002.

After land use maps of the study area in 1986 and 2002 were produced, the Kappa coefficient, one of the most popular measures [22], was used to make an accuracy assessment for land use classifications. Based on another reconnaissance field survey in July of 2002, the overall classification accuracy of the land use map for 1986 and 2002 was determined to be 83.2% and 86.7% respectively. And the Kappa coefficient for 1986 and 2002 maps was 0.77 and 0.82 respectively. The wrong identities of pixels were all corrected. The revised classification maps of land use in Lijiang County in1986 and 2002 were shown in Figure 3.

### 3.2 Methods

#### 3.2.1 Analysis of land use change matrix

To detect in detail the process of LUCC in Lijiang County during 1986-2002, an analysis of land use change matrix was conducted through the spatial overlay of the two land use maps shown in Figure 3. Generally speaking, the overlay of two land use maps A_i*i_ and B_i*i_ keeps to the map algebraic formula (1), which is valid with the number of land use types not more than 10. After the map algebraic operation, a map of land use change, C_i*j_ is derived to represent the types of land use change from time A to time B and associated spatial distribution.

(1) $${\text{C}}_{\text{i}*\text{j}}={\text{A}}_{\text{i}*\text{j}}*10+{\text{B}}_{\text{i}*\text{j}}$$

#### 3.2.2 Analysis of the rate of land use change

Two kinds of land use change rate were focused in this case study, i.e. the change rate of single land use type, and the integrated land use change rate for the whole region [58].

(1) Change rate of single land use type
  As land use dynamic degree for single land use type can quantitatively measure the change of a certain land use type, the index is recognized as one of the most widely used indices for detecting the land use change rate. It is mostly calculated according to equation (2):
  (2) $$K_{\text{1}}=\frac{U_{b}-U_{a}}{U_{a}}\times \frac{1}{T}\times 100\%$$
  where *K*_1_ is land use dynamic degree, measuring the change rate of the target land use type; *U_a_* and *U_b_* are the area of the target land use type at the beginning and end of the study period respectively; and *T* is the study period, which is usually measured with the unit of year.
  The index of *K*_1_ can concisely express the overall characteristics of the change of a certain land use type in the study period, while it is acknowledged that *K*_1_ has missed some information on the spatial process of land use change. Generally speaking, there are three change types for a certain land use type in the process of land use change, i.e. no conversion, conversion to other land use types, and conversion from other land use types. It is obvious that what is measured by the index of *K*_1_ is the integration of the three kinds of change for the target land use type in the study area, and it cannot express the information on each change type directly. Thus, another index for the change rate of single land use type was constructed according to equation (3):
  (3) $$K_{2}=\frac{\Delta in+\Delta \text{out}}{U_{a}}\times \frac{1}{T}\times 100\%$$
  where *K*_2_ is land use dynamic degree, measuring the change rate of the target land use type; Δ*in* and Δ*out* are the area of the target land use type conversion from or to other land use types in the study period respectively; and *T* is the study period, which is usually measured with the unit of year.
  Focusing on the process of land use change, the index of *K*_2_ can effectively reflect the area ratio of the conversion from and to the target land use type. However, it was criticized that *K*_2_ could not compare the conversion loss rate and conversion gain rate [59]. As what the index of *K*_1_ reflects includes the information on the comparison between conversion loss and gain, both *K*_1_ and *K*_2_ are used to measure the change rate of single land use type. In details, the index of *K*_2_ quantifies the total “loss or gain” conversion of the target land use type; while the index of *K*_1_ reflects the algebraic summation of conversion loss and gain of the target land use type, and the absolute value of *K*_1_ measures the extent of the relative difference between conversion loss and gain, with a positive sign of the value of *K*_1_ for the dominance of conversion gain from other land use types to the target land use types, and a negative sign for the precedence of conversion loss.
(2) Integrated land use change rate for the whole region
  In order to detect the change rate of regional land use as a whole, an index of integrated land use dynamic degree was used in this study, which was calculated according to equation (4):
  (4) $$\text{LUC}=\left[\frac{\overset{n}{\underset{i=1}{\text{∑}}}\Delta LU_{i\to j}}{LU}\right]\times \frac{1}{T}100\%$$
  where *LUC* is integrated land use dynamic degree, measuring the integrated land use change rate for the whole region; Δ*LU_i→j_* is the area converted from land use type *i* to land use type *j; n* is the number of land use types; *LU* is the total area of the study area; and *T* is the study period, which is usually measured with the unit of year.

#### 3.2.3 Analysis of the change of land use patterns

At present, the change of land use patterns was usually monitored and assessed with the application of landscape metrics. Landscape metrics are ecological indicators used to quantify the composition and spatial configuration of landscapes. Since they not only quantify spatial patterns, but also interpret ecological information hidden in spatial data, landscape metrics can correlate the ecological process and spatial patterns in a landscape. And it is the introduction and development of landscape metrics that have lead to the rapid development of landscape ecology since the 1990s [67]. Thus, as an essential approach in quantifying landscape spatial patterns with distinct ecological implications, landscape metrics is a great help in the analysis of changes in land use patterns and associated ecological effects.

In this study, six commonly used metrics were chosen for the analysis of landscape patterns, including Shannon's diversity index (SHDI) measuring landscape diversity, contagion index (CONT) and fragmentation index (FN) quantifying spatial configuration, and area-weighted mean patch fractal dimension (AWMPFD), mean patch size (MPS) and mean patch perimeter (MPP) detecting patch shape. The calculation and associated ecological meanings of these landscape metrics were detailed in the related studies [68-70]. After land use data had been converted to Arc Grid form, the software package, FRAGSTATS 3.3 was used to compute the selected landscape metrics.

## 4. Results

### 4.1 Land use change as a whole

As shown in Tables 1 and 2, although there has been no addition or disappearance of land use types, land use structure and patterns have changed significantly during the period of 1986-2002 in Lijiang County. Even though land use structure changed by only 8.26%, this change amounted to 213,435 ha. Waste grassland decreased more than twice as much as dense forest land and grassland; sparse forest land, paddy field, and dry land increased marginally more than construction land, bare land, and water body; and there was little increase of glacier or snow-capped land. However, the typical characteristics of rural land use in mountain areas hardly changed in Lijiang County, as represented by the clear dominance of dense forest land and a large proportion of sparse forest land, dry land and paddy field. From 1986 to 2002, the number of patches in the study area has increased 16.89% with 2,497 patches added, resulting in a distinct decrease of mean patch sizes and perimeters. While there were few changes in the general characteristics of spatial patterns of the agricultural landscape in Lijiang County, which was reflected in a low patch number and high mean patch size and perimeter. This was mainly due to limiting natural conditions and environmental protections in the study area. Located at the high, cold mountain areas in northwestern Yunnan Plateau, Lijiang County is characterized by deep gorges and fragmented terrain, where there has always been low population densities and a low intensity of human activities on the steep terrain. Thus, such natural vegetations as dense forest land and sparse forest land were highly conserved with large patches remaining in high latitude areas, especially after the initiation of China's Grain-for-Green Programme and Natural Forest Protection Project since 1998.

Among all the land use types, both the mean patch size and perimeter of dense forest land were the largest, with a distinct decrease during 1986-2002. The increase in small patches of dense forest land were mainly due to the conversion from dense forest land to other land use types, resulting in the fragmentation of large patches of dense forest land. Correspondingly, the total area of sparse forest land increased by 17.65% with a 29.65% increase of number of patches, while there was an increase of mean patch perimeter with the decrease of mean patch size. The change of patch characteristics of sparse forest land showed that, the increase in sparse forest land was mainly due to scattered patches converted from other land use types.

There was some increase of total area of paddy field, while its patch number doubled with a distinct decrease in mean patch sizes and perimeters. This could be explained as follows: there was some conversion loss of paddy field, which resulted in the fragmentation of its large patches by other landscape elements; on the other hand, newly converted paddy field could just been cultivated in scattered small patches with appropriate natural conditions of water and heat, due to rugged terrain in the study area. Meanwhile, there was a small increase of total area and decrease of patch number of dry land, with some increase in mean patch size and perimeter, which showed that newly formed dry land was mainly cultivated from the fringe of the former so as to merge as large patches.

Along with the decrease of total area and number of patches, there were increase of mean patch size and decrease of mean patch perimeter of grassland, suggesting that the grasslands lost were from small patches more than from large patches. It was on the contrary for the change of water body. There were a little increase of total area and great increase of number of patches, with a distinct decrease in mean patch size and perimeter. This meant that newly formed water bodies were mainly comprised of small patches. Due to special requirements for climate conditions, there was a limited spatial distribution of glacier or snow-capped land in Lijiang County, with little change in total area and number of patches in the study period. And all the glacier or snow-capped land aggregated in the peak of mountains, which usually formed large patches. This was the reason for its higher mean patch size and perimeter than that of other land use types except dense forest land.

The change of construction land showed a remarkable increase in total area, with some increase of number of patches, and mean patch size and perimeter of construction land, indicating that urban sprawl was the dominant driver increasing construction land. There was a decrease of total area, number of patches, and mean patch size and perimeter of waste grassland, partly due to the conversion of waste grassland both in large or small patches. And for bare land, its number of patches increased far more than total area, with a distinct decrease of mean patch size and perimeter. This suggested that, the newly formed bare land was mainly in small patches.

### 4.2 Land use change matrix during 1986-2002

As shown in Table 2, the process of change for each land use type was quite different, which could be characterized as follows:

- Glacier or snow-capped land, water body and construction land were the most stable land use types with a conversion loss less than 4% of their total area. Compared to little conversion gain of glacier or snow-capped land, there was a little conversion gain of water body evenly from other land use types except glacier or snow-capped land and construction land, and the total area of construction land was about doubled with a dominant conversion gain from paddy field;
- Dense forest land could also been regarded as a stable land use type with a area ratio of 85.64% unchanged in the study period, and sparse forest land was the main conversion destination. However, due to its dominant area ratio to the total area of the study area, all the conversions from dense forest land to other land use types were dominant in the conversion gain of corresponding land use types;
- Paddy field and dry land showed distinct changes with an area ratio of 40.67% and 58.62% during 1986-2002, respectively. Paddy field was mainly converted into dry land and construction land, while dense forest land was dominant in the conversion loss of dry land. The un-stability of about half of arable land, and the coexistence of abandon and cultivation of arable land, suggested the low soil quality and land productivity in the study area;
- Sparse forest land, bare land and grassland were more unstable with only 31.05%, 36.96% and 40.36% unchanged respectively, while their main conversion destinations were all dense forest land, which was partly due to the initiation of natural forest protection in the study area;
- Waste grassland changed the most during 1986-2002 with 25.74% unchanged, and most converted into dry land and dense forest land.

**Figure 4.**
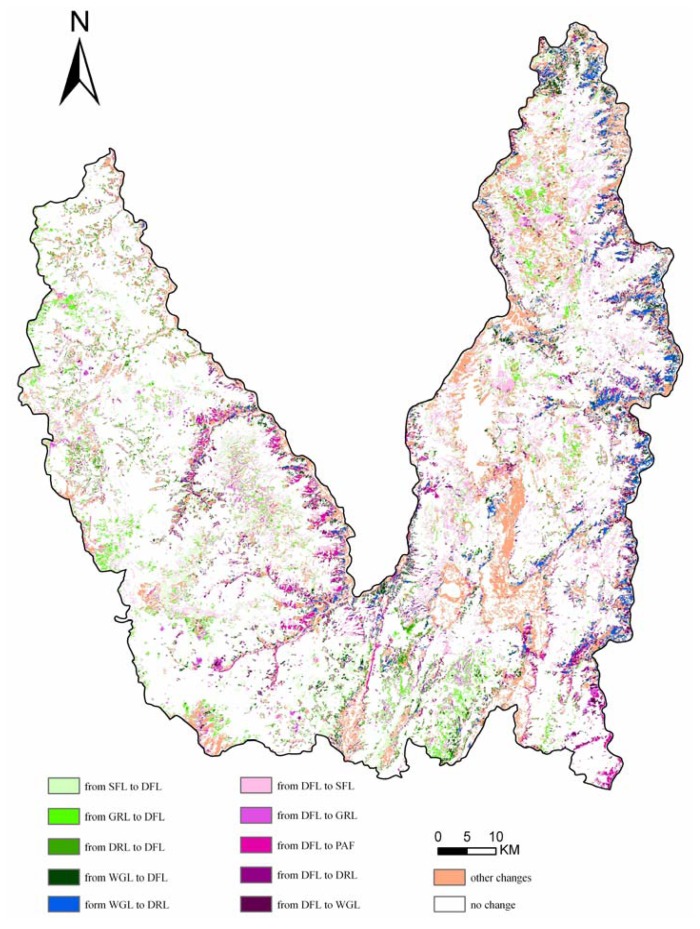
Dominant land use change processes in Lijiang County from 1986 to 2002.

Meanwhile, using greater than 1% of total area of the study area as the threshold for delineating significant processes of land use change in Lijiang County, ten important land use conversions were selected as follows (Figure 4): conversion from dense forest land to sparse forest land (4.35%, area ratio of total area of the study area, the same as following), conversion from sparse forest land to dense forest land (2.78%), conversion from grassland to dense forest land (2.06%), conversion from waste grassland to dry land (1.65%), conversion from dry land to dense forest land (1.45%), conversion from waste grassland to dense forest land (1.43%), conversion from dense forest land to dry land (1.40%), conversion from dense forest land to paddy field (1.13%), conversion form dense forest land to waste grassland (1.04%), and conversion from dense forest land to grassland (1.01%). Among these land use conversions, there was only one kind of conversion, i.e. the conversion from waste grassland to dry land, that had nothing to do with dense forest land, and the others were all related to the conversion loss or gain of dense forest land. Therefore, it could be concluded that, the key driving force of regional land use change in Lijiang County was the decrease and increase of the patches of dense forest land, which resulted from deforestation and afforestation or forest conservation, respectively.

### 4.3 Land use change rate during 1986-2002

#### 4.3.1 Change rate of each land use type

**Figure 5.**
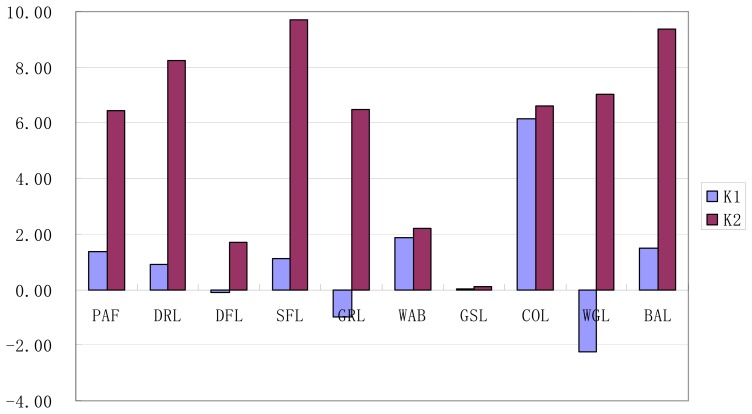
Change rate of single land use type in Lijiang County during 1986-2002.

As shown in Figure 5, on one hand, as to the index of *K*_1_ measuring annual net increasing rates of land use types, construction land scored the highest, with a value of 6.14%, followed in order by waste grassland, water body, bare land, paddy field, sparse forest land, grassland and dry land, while dense forest land and glacier or snow-capped land accounted the lowest with a value of -0.1% and 0.04%, respectively. On the other hand, as to the index of *K_2_* quantifying annual rates of conversion loss and gain of land use types, sparse forest land got the highest value of 9.72%, followed orderly by bare land, dry land, waste grassland, construction land, grassland, paddy field, water body and dense forest land, and glacier or snow-capped land was the lowest with a value of 0.11%.

In summary, taking notice of that the general characteristics of regional land use in the study area were represented as rural land use in western China, land use dynamic degree of each land use type in Lijiang County was a little high with quite differences during 1986-2002. Among the ten land use types, taking both indexes into account, glacier or snow-capped land had the lowest land use dynamic degree, followed by dense forest land and water body; while the dynamic degrees of the other land use types were relatively high, with sparse forest land for the highest.

According to the difference of the indexes of *K*_1_ and *K*_2_, dynamic degree of all the land use types in Lijiang County could be classified into five types, and focusing on area ratio to the total area of the whole county in 1986, their corresponding conversion characteristics were also shown as follows:

- Type I with high annual changing rate and high net increasing rate, only including construction land. The change of construction land was characterized as a great deal of conversion gain with little conversion loss;
- Type II with high annual changing rate and low net increasing rate, including paddy field, dry land, sparse forest land and bare land. Their land use change was represented as a great conversion loss with a little more conversion gain;
- Type III with low annual changing rate and low net increasing rate, including water body and glacier or snow-capped land, which had a little conversion gain and little conversion loss;
- Type IV with high annual changing rate and low net decreasing rate, including grassland and waste grassland. There was a great conversion loss with a little less conversion gain;
- Type V with low annual changing rate and low net decreasing rate, only including dense forest land, which had a little conversion gain with some more conversion loss.

#### 4.3.2 Integrated change rate of sub-regional land use

As shown in Table 3, there was a certain regional difference of integrated land use change rate among the 24 towns in Lijiang County. Dayan Town had the highest change rate of 3.34% per year, in contrast with the lowest change rate of 0.98% per year in Tacheng Town. Applying the statistic analysis software of SPSS16.0 for windows, a hierarchical cluster analysis with the cluster method of between-groups linkage was conducted on the integrated regional land use change rate of all the 24 towns. The results showed that, all the 24 samples could be classified into five types: (1) Towns I with the highest integrated land use change rate, only including Dayan Town; (2) Town II with higher integrated land use change rate, including two towns; (3) Town III with moderate integrated land use change rate, including 14 towns; (4) Town IV with lower integrated land use change rate, including four towns; and (5) Town V with the lowest integrated land use change rate, including three towns.

To make certain the major influencing factors concerning the integrated land use change rate, applying the statistic analysis software of SPSS16.0 for windows, the analysis of bivariate correlations was conducted with the application of Pearson correlation coefficient. Three factors were chosen in the analysis of bivariate correlations, i.e. mean altitude, population density, and GDP per unit area (Table 3). The results showed that, all the three bivariate correlations between each influencing factor and integrated land use change rate had passed the 0.01 significance test, while the correlation coefficients were a little low. GDP per unit area and population density had positive moderate correlation with the correlation coefficient of 0.590535 and 0.588937 respectively, and mean altitude was thought to be irrelevant with the integrated land use change rate with the correlation coefficient of -0.15917.

### 4.4 Land use pattern change during 1986-2002

#### 4.4.1 Change of landscape diversity

Reflecting the richness and area evenness of landscape elements, Shannon's diversity index (SHDI) was widely used in detecting the change of landscape diversity. Generally speaking, a higher value of SHDI in a landscape refers to more landscape elements or more evenness of area ratio among all the landscape elements. In the study period, the value of SHDI in the whole county was a little low with some increase from 1.2401 to 1.2833. As there was no increase or decrease of landscape elements during 1986-2002, it seemed that there was a great and slightly decreasing unevenness in the area ratio of landscape elements.

The change of SHDI can be explained by the dynamics of landscape structure. On one hand, dense forest land was dominant in the whole study period with an area ratio over 67%; on the other hand, from 1986 to 2002, along with the shrinkage of dense forest land, grassland and waste grassland, all the other land use types had experienced a small increase. Therefore, there was a tendency of balanced landscape structure in the process of landscape change.

#### 4.4.2 Change of spatial configuration

Contagion index (CONT) was used to quantify the juxtaposing and clumping of landscape elements in the whole landscape. Generally speaking, the value of CONT is between 0 and 100, and a high value means that the landscape is composed of a few aggregated large patches, with a low value referring to the dominance of a lot of disaggregated and interspersed small patches. In the study period, there was a moderate value of CONT from 64.8628 to 63.5813 in the study area, which might result from the slight aggregation of large patches of dense forest land and glacier or snow-capped land; while the decrease of CONT in the study period indicated the weakening of the aggregation extent of those large patches.

Fragmentation indexes (FN) at landscape level and class level are both calculated to quantify human disturbances on the rural landscape in Lijiang County. In the study period, there was a distinct increase of FN from 0.019775 to 0.023115, suggesting the strengthening of human disturbances in the process of landscape change, which might partially result from the rapid development of tourism and associated environmental impact by tourists in the study area.

**Figure 6.**
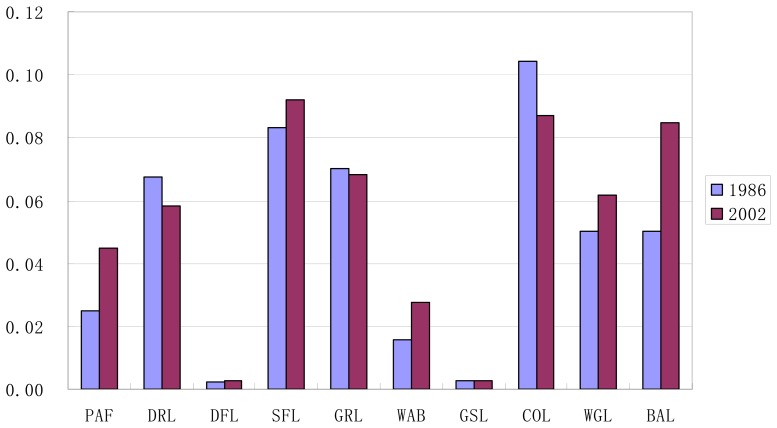
Change of FN at class level in Lijiang County during 1986-2002.

Through Figure 6, it could be concluded that there was remarkable difference among FN at class level. From 1986 to 2002, the FN of construction land and sparse forest land were always the highest, with glacier or snow-capped land and dense forest land the lowest, which accorded with their difference in human disturbances. In details, the changes of FN of all the ten landscape elements in the study period were as follows: (1) along with the increase of area ratio, there was an increase of the FN of paddy field, sparse forest land, water body and bare land, which indicated that the conversion gain areas of these land use types mainly came from scattered small patches, and few bordered on former patches with the same land use type to form large patches; (2) the FN of grassland decreased with the shrinkage of area ratio, which meant that conversion loss of grassland was mainly in the form of small patches; (3) the FN of dense forest land and waste grassland increased in contrast with the decrease of their area ratio, which suggested that some aggregated large patches might be divided into scattered small patches in the process of conversion loss; and (4) the FN of construction land, dry land and glacier or snow-capped land decreased together with the increase of their area ratio, which showed that newly conversion gain from other land use types mainly resulted from the extending of former patches of corresponding land use types, and aggregated large patches came into being through the amalgamation of newly formed and former patches with the same land use type as a result.

What should be stressed was that, as two land use types with similar human disturbances, both paddy field and dry land experienced the increase of area ratio, while the changes of their FN in the study period were on the contrary. This could be explained by the terrain characteristics of the study area and the natural restrictions of spatial distribution of paddy field and dry land. In contrast with dry land, the cultivation of paddy field was strictly restricted by natural environment, i.e. flat terrain, low relative relief and abundant irrigation water. However, distributing high mountains and deep gorges, Lijiang County was characterized with fragmented terrain, and there were no tracks of land suitable for paddy field after thousands years of agricultural cultivation, which leaded to the increase of paddy field in the study period must be in the form of scattered small patches and could not be amalgamated with former patches of paddy field to form large patches. On the other hand, as there was no such natural restriction on the cultivation of dry land, the newly formed dry land mainly distributed among former small patches with the application of agricultural intensive operation. Thus the increase of dry land experienced the process of amalgamation of scattered small patches and associated formation of aggregated large patches, which resulted in the decrease of patch number along with the increase of area ratio of dry land, and the decrease of FN of dry land at last.

#### 4.4.3 Change of patch shape

The index of fractal dimension was widely used to quantify the complexity of patch shape at a given observation scale. As the index of area-weighted mean patch fractal dimension (AWMPFD) takes both patch fractal dimension and patch area into account, what it mainly reflected is the shape complexity of large patches, and it is more appropriate than the index of fractal dimension to quantify the change of landscape patterns in view of patch shape. Generally speaking, the lower the value of AWMPFD is, the more regular patch shape in the landscape is, and the greater human disturbance on the patches is, since human activities usually lead to regular geometrical shape of patches.

**Figure 7.**
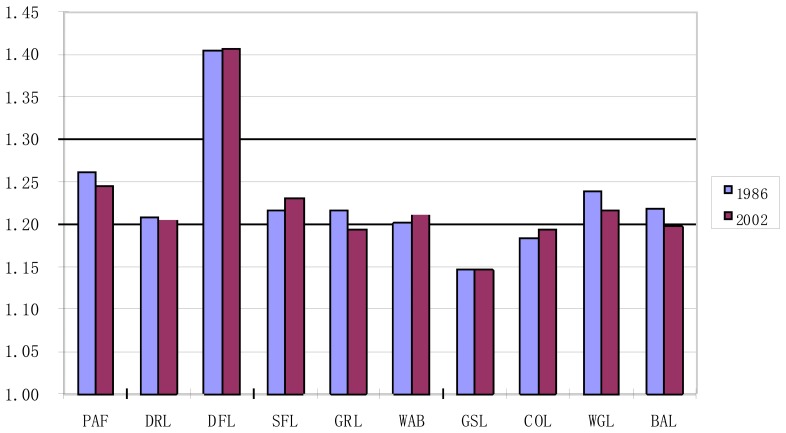
Change of AWMPFD at class level in Lijiang County during 1986-2002.

As shown in Figure 7, among all ten kinds of land use types, AWMPFD of dense forest land was always the highest during the study period, while construction land and glacier or snow-capped land had the lowest ones. This was mainly due to high human disturbances on construction land and low human activities in dense forest land. And because the spatial distribution of glacier or snow-capped land was highly restricted by climate conditions, it mainly distributed in mountain areas with the altitude over 4,250m, and extended from the peak to snow line. Thus, the patch shapes of glacier or snow-capped land were usually the same as a circle arc with the peak as the center of the circle. These regular geometric shapes would un-doubtfully result in the low value of AWMPFD.

Furthermore, as there were distinct decreases of AWMPFD of paddy field, dry land, grassland, glacier or snow-capped land, waste grassland and bare land, it suggested the increasing of the intensity of human activities in these land use types; while the increases of AWMPFD of sparse forest land, dense forest land and water body meant the decreasing of human disturbances; and it might be the increasing of disorder of human disturbances that resulted in the increase of AWMPFD of construction land, since construction land was highly affected by human activities.

## 5. Conclusions

It is widely acknowledged that land use/land cover change is a local environmental issue with global importance [11]. As the first step of a full LUCC research to provide primary data for the latter studies, the detecting of land use change is of great importance, which includes the monitoring of land use change in quantitative structure and spatial patterns. However, compared with the flourish of studies on the change of regional land use in quantitative structure, few studies have focused on the analysis of the change of land use patterns.

Taking Lijiang County of Yunnan Province as a case study area, the study reported in this paper made an integrated analysis on the change of rural land use structure and patterns in mountain areas of western China during 1986-2002, using remote sensing and geographic information system techniques. The results showed significant land use change in quantitative structure and spatial patterns, although the typical characteristics of rural land use in mountain areas hardly changed in Lijiang County, which were represented as absolute dominance of dense forest land and a large proportion of sparse forest land, dry land and paddy field, and a low patch number and high mean patch size and perimeter. In details, the increase of construction land, paddy field and dry land, together with the decrease of dense forest land and waste grassland were the key issues of rural land use change, and the conversions between dense forest land and sparse forest land, grassland, waste grassland and dry land were the primary land use change processes. In the study period, glacier or snow-capped land had the lowest land use dynamic degree, with sparse forest land for the highest; while there was distinct regional difference of integrated regional land use change rate among the 24 towns of Lijiang County, which mainly resulted from human settlement and rural economic development and was statistically irrelevant with environmental conditions.

Quantified through landscape metrics, the change of land use patterns in the study area was characterized as the increase of landscape diversity and landscape fragmentation, and the regularization of patch shapes, suggesting the intensification of edge effects, the decrease of landscape stability, and associated degradation of ecological quality in the rural landscape. Although far more additional data are needed to make certain the driving forces for the increased fragmentation, urban sprawl is regarded to be a great contributor. There was distinct decrease in the mean patch sizes of paddy field and dense forest land, with the increase of construction land (Table1). In combination with Table 2, it could be concluded that many paddy field areas in 1986 were converted to urban areas in 2002, while this was compensated by conversion from forest lands to paddy field. It seemed that paddy fields were occupied in the process of urban sprawl, and farmers were forced to start new paddy fields in remote areas.

Nevertheless, what should be pointed out is the classification accuracy of remote sensing images, which is critical to reliable detecting of land use change [71-73]. As we know, the rugged terrain is a great obstacle to the image classification and associated accuracy survey; when the actual land use changes are small, they might fall within the errors for classification. In the case study area, as stated above, there are only 4.7% of basin, with the other for mountains and valleys; while compared with land use change in eastern China, the annual change rate of 0.52% is far too small. Although the land use classification has passed the overall accuracy test, it is undoubted that some changes might be hidden by classification errors. As the use of remote sensing data will continue to grow in the analysis of LUCC, it is necessary to apply other remote sensing data with higher resolution besides the Landsat TM images, so as to reduce the influence of classification errors to further detailed studies on LUCC in mountain areas.

Furthermore, in order to make a full depiction of the change of rural land use in the study area, future studies are needed to be focused on the driving forces analyzing, change modeling and predicting, and the ecological effects assessing of land use change. In addition, what should be stated is the representativeness of Lijiang County for remote regions in western China. Obviously, the study area cannot represent the whole western China, since western China has about 56.4% area of the nation with great regional difference. However, as stated above, Lijiang County is a typical rural minority area with fragile environment and low economic development in mountain areas of western China. Thus, to reflect a broad and full picture of rural land use change in western China, it is necessary to compare this case study with other counties in western China under different economic development models, cultural traditions, or natural environmental conditions.

## Figures and Tables

**Table 1.** The change of number of patches, area ratio, and mean patch size and perimeter in different land use types in Lijiang County during 1986-2002.

**Land use type**	**Number of patches**		**Area ratio (%)**		**Mean patch size(hm^2^)**		**Mean Patch Perimeter (km)**	

	**1986**	**2002**	**1986**	**2002**	**1986**	**2002**	**1986**	**2002**
PAF	750	1,633	3.99	4.86	39.73	22.25	5.8411	4.0144
DRL	2,751	2,713	5.44	6.21	14.79	17.12	3.4388	3.6950
DFL	1,108	1,275	68.56	67.51	462.65	395.91	27.3465	23.7128
SFL	3,848	4,989	6.17	7.26	12.00	10.88	3.4942	3.6546
GRL	2,450	2,019	4.68	3.95	14.28	14.63	3.5825	3.2814
WAB	83	188	0.70	0.91	62.8	36.06	6.9958	4.0395
GSL	23	23	1.17	1.18	380.79	383.78	10.7739	10.693
COL	536	887	0.69	1.37	9.57	11.47	2.9125	3.0669
WGL	2,465	1,930	6.54	4.19	19.85	16.23	4.1566	3.5522
BAL	772	1,626	2.06	2.56	19.92	11.79	4.3692	3.0796

**Table 2.** Land use change matrix in Lijiang County during 1986-2002 (ha).

**Land use type in 1986**	**Land use type in 2002**										

	**PAF**	**DRL**	**DFL**	**SFL**	**GRL**	**WAB**	**GSL**	**COL**	**WGL**	**BAL**	**Total**
PAF	**18,104**	3,265	1,703	427	162	406	0	3,009	2,051	1,388	30,515
DRL	3,848	**17,215**	11,059	3,341	2,205	104	0	466	2,713	653	41,604
DFL	8,652	10,697	**449,050**	33,296	7,708	367	0	524	7,970	6,082	524,346
SFL	2,402	2,935	21,230	**14,652**	1,340	175	0	429	3,662	363	47,188
GRL	11	573	15,745	1,263	**14,445**	104	0	0	1,156	2,495	35,792
WAB	17	20	18	2	1	**5,212**	0	31	22	30	5,353
GSL	0	0	13	7	2	2	**8,902**	0	3	21	8,950
COL	89	18	33	1	0	3	0	**5,087**	34	13	5,278
WGL	4,011	12,620	10,944	1,996	3,816	360	0	700	**12,875**	2,696	50,018
BAL	63	180	6,510	533	544	216	110	213	1,564	**5,823**	15,756
Total	37,197	47,523	516,305	55,518	30,223	6,949	9,012	10,459	32,050	19,564	764,800

Note: The unchanged area proportion of each land use type was marked in bold.

**Table 3.** Integrated land use change rate and associated influencing factors in different towns of Lijiang County during 1986–2002.

**Township**	**LUC**	**Population Density (Person/Km^2^)**	**GDP per unit area (10^3^ RMB/Km^2^)**	**Mean altitude (M)**	**Township**	**LUC**	**Populatin Density (Person/Km^2^)**	**GDP per unit area (10^3^ RMB/Km^2^)**	**Mean altitude (M)**
Baisha	1.95	34.84	51.47	3,089.46	Lashi	1.94	110.98	223.80	2,691.51
Baoshan	2.63	19.43	20.28	3,033.73	Liming	1.27	14.80	20.35	3,034.76
Dadong	1.84	25.23	29.87	2,806.08	Longpan	1.82	36.00	62.45	3,014.71
Daju	2.17	21.21	50.34	2,990.7	Longshan	1.92	38.07	67.29	2,563.16
Dayan	3.34	4,564.47	7,084.01	2,477.06	Ludian	1.42	32.93	50.08	3,047.82
Fengke	2.60	22.66	23.27	2,615.18	Mingyin	2.05	23.04	26.10	2,565.85
Huangshan	1.80	101.59	318.90	2,727.11	Qihe	1.87	51.70	113.84	2,734.46
Jinjiang	1.28	45.73	61.87	2,133.98	Renhe	1.41	11.93	12.33	3,019.07
Jinshan	2.06	82.20	151.96	2,563.59	Shigu	2.22	52.75	134.93	2,640.82
Jinzhuang	1.89	39.66	102.97	2,607.28	Shitou	1.10	15.74	25.64	2,981.85
Jiuhe	1.67	78.84	115.43	2,661.21	Tacheng	0.98	34.09	56.98	2,770.73
Judian	0.99	53.20	94.85	2,636.71	Tai'an	1.72	31.27	35.82	2,830.62
